# A Comprehensive Genomics Solution for HIV Surveillance and Clinical Monitoring in Low-Income Settings

**DOI:** 10.1128/JCM.00382-20

**Published:** 2020-09-22

**Authors:** David Bonsall, Tanya Golubchik, Mariateresa de Cesare, Mohammed Limbada, Barry Kosloff, George MacIntyre-Cockett, Matthew Hall, Chris Wymant, M. Azim Ansari, Lucie Abeler-Dörner, Ab Schaap, Anthony Brown, Eleanor Barnes, Estelle Piwowar-Manning, Susan Eshleman, Ethan Wilson, Lynda Emel, Richard Hayes, Sarah Fidler, Helen Ayles, Rory Bowden, Christophe Fraser, HPTN 071 (PopART) Team

**Affiliations:** a Big Data Institute, Li Ka Shing Centre for Health Information and Discovery, Nuffield Department of Medicine, University of Oxford, Oxford, United Kingdom; b Wellcome Centre for Human Genetics, University of Oxford, Oxford, United Kingdom; c ZAMBART, University of Zambia, Lusaka, Zambia; d London School of Hygiene and Tropical Medicine, London, United Kingdom; e Peter Medawar Building for Pathogen Research, University of Oxford, Oxford, United Kingdom; f Dept. of Pathology, Johns Hopkins University School of Medicine, Baltimore, Maryland, USA; g Statistical Centre for HIV/AIDS Research, Fred Hutchinson Cancer Research Centre, Seattle, Washington, USA; h Department of Infectious Disease, Imperial College London, Imperial College NIHR BRC, London, United Kingdom; Rhode Island Hospital

**Keywords:** HIV, NGS, viral genomics, public health, sub-Saharan Africa, viral sequencing, bait capture, short-read sequencing, Illumina, SMARTer, HPTN, PopART, HPTN 071, phylogenetics, viral evolution, drug resistance, antiretroviral therapy, RNA virus, antiretroviral resistance, drug resistance evolution, gene sequencing, human immunodeficiency virus, phylogenetic analysis, surveillance studies

## Abstract

Viral genetic sequencing can be used to monitor the spread of HIV drug resistance, identify appropriate antiretroviral regimes, and characterize transmission dynamics. Despite decreasing costs, next-generation sequencing (NGS) is still prohibitively costly for routine use in generalized HIV epidemics in low- and middle-income countries. Here, we present veSEQ-HIV, a high-throughput, cost-effective NGS sequencing method and computational pipeline tailored specifically to HIV, which can be performed using leftover blood drawn for routine CD4 cell count testing. This method overcomes several major technical challenges that have prevented HIV sequencing from being used routinely in public health efforts; it is fast, robust, and cost-efficient, and generates full genomic sequences of diverse strains of HIV without bias. The complete veSEQ-HIV pipeline provides viral load estimates and quantitative summaries of drug resistance mutations; it also exploits information on within-host viral diversity to construct directed transmission networks. We evaluated the method’s performance using 1,620 plasma samples collected from individuals attending 10 large urban clinics in Zambia as part of the HPTN 071-2 study (PopART Phylogenetics). Whole HIV genomes were recovered from 91% of samples with a viral load of >1,000 copies/ml. The cost of the assay (30 GBP per sample) compares favorably with existing VL and HIV genotyping tests, proving an affordable option for combining HIV clinical monitoring with molecular epidemiology and drug resistance surveillance in low-income settings.

## Abstract (precis)

Viral genetic sequencing can be used to monitor the spread of HIV drug resistance, identify appropriate antiretroviral regimes, and characterize transmission dynamics. Despite decreasing costs, next-generation sequencing (NGS) is still prohibitively costly for routine use in generalized HIV epidemics in low- and middle-income countries. Here, we present veSEQ-HIV, a high-throughput, cost-effective NGS sequencing method and computational pipeline tailored specifically to HIV, which can be performed using leftover blood drawn for routine CD4 cell count testing.

## INTRODUCTION

Achieving sustained reductions in the incidence of HIV infections through programs of universal access to testing and antiretroviral treatment (UTT) remains a major goal in public health. International efforts have been focused on working toward the UNAIDS “90-90-90” targets, with 90% of people living with HIV (PLWH) diagnosed, 90% of those on antiretroviral therapy (ART), and 90% of those successfully virally suppressed (1, 2). HIV drug resistance compromises the ability of ART to suppress viral replication. The frequency of drug resistance is expected to increase as UTT becomes more common (3), which may make it difficult to reach the WHO goal. A 2017 report by the WHO identified parts of the world where more than 10% of people living with HIV already harbor virus resistant to current first line antiretroviral drugs (4). This has driven the switch to dolutegravir-based regimens as preferred first line ART.

Both the spread of drug resistance and transmission patterns can be better understood by analyzing viral sequence data (5). To date, clinical drug resistance testing has primarily relied on Sanger consensus sequencing of HIV *pol* genes. Next-generation sequencing (NGS) also produces detailed minority variant information, which can detect low-frequency drug resistant viral variants. However, despite its benefits, adoption of NGS for HIV drug resistance testing has been slow, in part due to technical difficulties in obtaining whole-genome sequences for all genotypes, particularly at low viral loads, and uncertainty over distinguishing low-frequency mutations from the sequencing artifacts and contamination that occur during massive parallel sequencing. Recently, the FDA approved the first NGS assay for HIV drug resistance using *pol*-specific PCR that can sequence up to 15 samples in parallel (6); however, demand remains for more high-throughput, low-cost options for use clinically and as a surveillance tool in high-prevalence settings. In addition, within an appropriate ethical framework, NGS enhances resolution in transmission analyses, indicating transmission direction and thus revealing population characteristics of transmitters and recipients (7). The potential for viral whole-genome sequencing to transform global health surveillance operations has been noted (8).

Large-scale NGS sequencing of HIV genomes using virus-specific PCR (9) has been used to produce whole viral genomes for European samples (10), but the method’s performance was found to be far from optimal for analysis of sub-Saharan African samples, with amplification failures resulting in biased genome coverage (11). We previously described veSEQ, a probe-based enrichment method, free of virus-specific PCR, which can be used to sequence viruses directly from clinical samples (12). Here, we describe veSEQ-HIV, a comprehensive laboratory and computational protocol specifically developed to support clinical management and public health programs in low-income settings.

## MATERIALS AND METHODS

### Samples.

Patients were recruited to the HPTN 071-2 (PopART phylogenetics) study by research assistants at 10 urban primary health care facilities, located in 9 of the 12 Zambian communities of the main trial (one community had two health care facilities) (13). The nine communities involved were evenly split between the three study arms of HPTN-071. Patients were recruited if they were aged 18 or over, not currently taking ART, and if they specifically consented to the ancillary phylogenetic study. Most patients were either newly enrolled in the clinic or enrolled and newly eligible for ART; a small fraction was recruited having recently missed several doses of ART. The study protocol (https://www.hptn.org/sites/default/files/inline-files/HPTN%20071-2%2C%20Version%202.0%20%2807-14-2017%29.pdf) has been approved by the ethics committees of the University of Zambia (c/o the Zambian ministry of health) and of the London School of Hygiene and Tropical Medicine.

### Sampling.

No additional blood samples were required for this ancillary study. Unused samples of blood collected from consenting individuals undergoing routine CD4 cell count testing were transported to the local hospital on the same day. Blood was centrifuged twice and two 500-μl aliquots of plasma were frozen at −80°C. Samples were transported to a central research laboratory (ZAMBART facility) in Lusaka, Zambia using a mobile −20°C freezer, and then shipped to the sequencing laboratory in the United Kingdom. Samples were processed approximately in order of collection and represented the diversity of the population recruited at the beginning of the study.

### Laboratory methods.

Total RNA was extracted with magnetized silica from HIV-infected plasma lysed with guanidine thiocyanate and with ethanol washes and elution steps performed using the NUCLISENS easyMAG system (bioMérieux). The total 30 μl elution volume was reduced with Agencourt RNAClean XP (Beckman Coulter) to maximize the input RNA mass while minimizing volume for library preparation.

Libraries retaining directionality were prepared using the SMARTer Stranded Total RNA-Seq kit v2 - Pico Input Mammalian (Clontech, TaKaRa Bio) with the following protocol specifications. Total RNA was first denatured at 72°C with the addition of tagged random hexamers to prime reverse transcription, followed by cDNA synthesis according to the manufacturer’s protocol option with no fragmentation. The first strand cDNA was then converted into double-stranded dual-indexed amplified cDNA libraries using in-house sets of 96 i7 and 96 i5 indexed primers (14), using a maximum of 12 PCR cycles. All reaction volumes were reduced to one quarter of the SMARTer kit recommendation and set up was either prepared manually or automated using the Echo 525 (Labcyte) low-volume liquid handler.

No depletion of ribosomal cDNA was carried out prior to target enrichment. Equal volumes (5 μl from a total of 12.5 μl) of each amplified library were pooled in 96-plex without prior cleanup. The pool was cleaned with a lower ratio of Agencourt AMPure XP than recommended by the SMARTer protocol, to eliminate shorter libraries (0.68×). The size distribution and concentration of the 96-plex was assessed using a High Sensitivity D1000 ScreenTape assay on a TapeStation system (Agilent) and a Qubit dsDNA HS Assay (Thermo Fisher Scientific).

A total of 500 ng of pooled libraries was hybridized (SeqCap EZ reagent kit, Roche) to a mixture of custom HIV-specific biotinylated 120-mer oligonucleotides (xGen Lockdown Probes, Integrated DNA Technologies), then pulled down with streptavidin-conjugated beads as previously reported (12). Unbound DNA was washed off the beads (SeqCap EZ hybridization and wash kit, Roche), and the captured libraries were then PCR amplified to produce the final pool for sequencing using a MiSeq (Illumina) instrument with v3 chemistry for a read length up to 300 nt paired-end. Alternatively, up to 384 samples were sequenced on HiSeq 2500 set to Rapid run mode using HiSeq Rapid SBS kit v2 with maximum read lengths of 250 nt.

To confirm assay quantivity, clinical viral load measurements were obtained for 146 specimens also sequenced with veSEQ-HIV. Oxford University Hospital’s clinical microbiology laboratory used the COBAS AmpliPrep/COBAS TaqMan HIV1 Test (Roche Molecular Systems, Branchburg, NJ, USA).

### Computational pipeline.

Raw sequencing reads were first processed with Kraken (15) to identify human and bacterial reads. Kraken was run with default parameters (k = 31 with no filtering), using a custom database containing the human genome together with all bacterial, archaeal and viral genomes from RefSeq, a subset of fungal genomes, and all 9,049 complete HIV genomes from GenBank (last updated 18 May 2018). Reads were filtered to retain only viral and unclassified sequences, and these were trimmed to remove adaptors and low-quality bases using Trimmomatic (16), retaining reads of at least 80 bp. Filtered, trimmed sequences were assembled into contigs using SPAdes (17) and metaSPAdes (18) with default parameters for k (21 to 127). Contiguous sequences assembled from both assembly runs were clustered using cd-hit-est to remove redundant contigs (19), retaining the longest sequence in each cluster with a minimum sequence identity threshold of 0.9. Contigs, together with the filtered reads, were then used to generate HIV genomes and variant frequencies using *shiver* (20), with position-based deduplication of reads enabled. Samples for which no contigs could be assembled were mapped to the closest known HIV reference as identified by Kallisto (21), hashing the filtered reads against a set of 199 HIV reference genomes from the Los Alamos HIV database (http://www.hiv.lanl.gov/), and taking the closest matching genome as the mapping reference for *shiver*. veSEQ-HIV is quantitative, in that the total amount of sequences recovered correlated with viral load. This arises because PCR conditions remain nonsaturating and unbiased probes are used for virus enrichment. A further slight improvement is obtained by computationally removing duplicate copies of viral fragments from sequence data, which are generated by non-virus-specific PCR steps in the protocol. The sequence-derived viral load, in copies/ml, was calculated from the number of deduplicated HIV reads for each sample, using a linear regression model derived from a subset of 146 samples for which we obtained an independent, clinically measured viral load. The *R*^2^ value for this model was 0.89, with no evidence of bias and a mean squared error of prediction of 0.324 log_10_ copies/ml. The model was used to estimate a sequence-derived viral load for the full data set.

A panel of quantification standards was used to ensure quantitativity and guard against batch effects. The standards comprised five dilutions of subtype B virus spiked into plasma (AcroMetrix HIV-1 Panel copies/ml, Thermo Fisher Scientific), and either one or two negative plasma controls. These were grouped with each batch of 90 HPTN 071-2 (PopART phylogenetics) samples at the point of RNA extraction. We first introduced these standards in batch 6, and have been using these to monitor the quantitativity of each batch.

Contaminant reads were identified and removed using *phyloscanner* for in-depth analyses of *pol* sequencing data. *Phyloscanner* contains several procedures not only for detecting contaminant reads in NGS data sets (7), but also for “blacklisting” them (specifically removing them from consideration for further analysis). Blacklisting works by identifying reads in a sample that are either (i) identical to those from a second sample but present in much smaller numbers, or (ii) are phylogenetically distant from the majority of the sample’s reads and are relatively few in number. A total of 373 overlapping genomic windows, each of length 340 bp, were selected, staggering the starting positions by 5 bp. For each 340-bp window, a phylogeny was inferred for all read pairs that fully spanned that window, and ancestral state reconstruction divided the reads for each sample into distinct groups (subgraphs), with the *phyloscanner* Sankoff k parameter set to 12.5. A group of reads was flagged as likely contamination if it contained three or fewer reads, or less than 0.1% of the total number of reads from the sample in that window. The consensus sequence and minority base frequencies were then recalculated from the resulting cleaned mapped reads using *shiver* (20). The complete workflow is included within *phyloscanner* (“phyloscanner clean”). “Phyloscanner clean” can be further optimized where the approximate proportion of expected contaminant reads is known, e.g., from laboratory controls.

Finally, both the consensus sequence and the cleaned reads were analyzed with the Stanford drug resistance tool (22) to determine consensus and minority drug resistance levels. Aggregated drug resistance predictions, accounting for mutations linked on the same read pair, were calculated as the maximum level of resistance (susceptible < potential low-level < low-level < intermediate < high-level) observed in at least 20% of merged read pairs spanning each position.

## RESULTS

**FIG 1:**
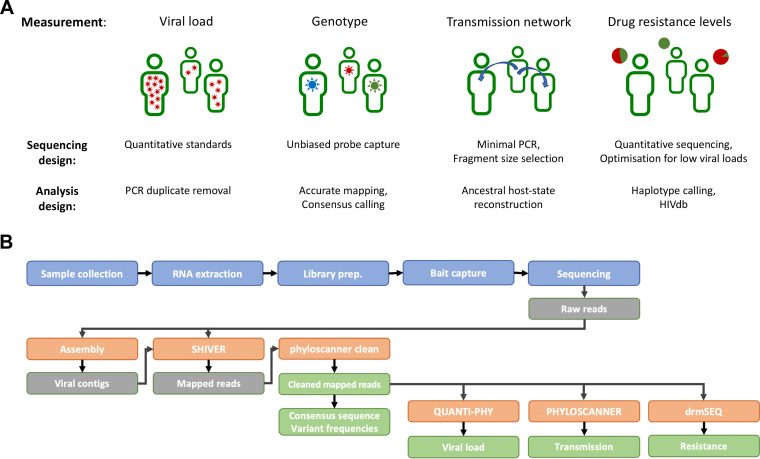
veSEQ-HIV includes a sequencing protocol and bioinformatics pipeline, yielding information on individual and population levels. (A) The veSEQ-HIV method was developed to provide multiple measurements from a single assay, including viral load, HIV genotype, drug resistance, and transmission inference. (B) Overview of veSEQ-HIV: a complete laboratory and computational pipeline for high-throughput sequencing. RNA extraction from plasma samples is carried out in a CL-3 certified laboratory, before transfer to a dedicated genomics facility for library preparation, bait capture, and finally sequencing. Raw sequencing data are preprocessed to remove host and contaminant RNA, and these computationally filtered reads together with their *de novo*-assembled contigs are used to determine the consensus genome and minority variant frequencies using *shiver*. QC metrics are then calculated, and the proportion of contaminant reads originating from other samples is estimated with Kallisto. Samples which result in a successful read mapping are then cleaned with *phyloscanner* to remove contaminant reads, and clean reads are used to infer transmission patterns with *phyloscanner*, and to make drug resistance predictions with HIVdb and drmSEQ.

The veSEQ-HIV protocol was developed to obtain multiple measurements from a single assay (Fig. 1). It provides a quantitative viral load estimate across at least 5 orders of magnitude, frequency of drug resistance mutations at both consensus and minority variant levels, and accurate and unbiased genotype information that is suitable for ancestral state reconstruction and the generation of directed transmission networks.

The method is the integration of a laboratory protocol with a bioinformatics pipeline (Fig. S1 in the supplemental material). Briefly, RNA extraction is followed by library preparation, bait capture, and sequencing. The bioinformatics pipeline removes host and contaminant RNA, and constructs consensus genome and minority variant frequencies from *de novo* assembled contigs using *shiver* (20). *Phyloscanner* is used to remove contaminant reads and infer transmission patterns (7). Drug resistance predictions are made with reference to HIVdb (Stanford database) (22).

### veSEQ-HIV is robust and cost-effective.

The development of veSEQ-HIV was achieved by optimizing veSEQ, our sequencing method for hepatitis C virus (HCV) (12). Our aims were to increase sensitivity and throughput, while minimizing cost, processing time, and protocol complexity. Compared to the enzymatic method for adapter ligation used in the original veSEQ protocol, the SMARTer protocol (Switching mechanism at 5′ end of RNA template) produced more unique (PCR deduplicated) sequences per sample, required fewer protocol steps and disposable plastics, and required no pre-PCR buffer exchanges (23). By concentrating extracted nucleic acids, (RNAClean XP) SMARTer reagent volumes could be reduced 4-fold without loss in library complexity. Automation was achieved in 96- and 384-well formats using 96-channel pipettes (PlateMaster, Gilson). The steps of the final protocol are listed in Table S1.

Like most high-throughput NGS protocols, veSEQ-HIV requires fragmentation of the virus RNA into so-called “inserts.” In previous work, we found that inserts of 350 bp or more offer useful insights into within-host phylogenetic diversity (7); we therefore sought to optimize the length of these inserts to be as long as possible within the limits compatible with the Illumina sequencing platform (350 to 600bp). After reducing preenrichment PCR cycles from 18 to 12 and introducing a size-selective bead cleanup to remove shorter fragments, over 40% of inserts within each sequencing library were in the desirable size range (Fig. S1).

Contamination can be physically introduced in the laboratory or occur due to index misassignment errors during sequencing, resulting in a number of reads being incorrectly attributed to a sample. The presence of these contaminant reads can undermine several important inferences: estimations of viral load (in particular distinguishing low viral load from aviraemia), detection of drug resistant minor variants, and the inference of transmission direction using within-host phylogenetics. We identified and blacklisted contaminant reads using the previously described routine “phyloscanner clean” in the *phyloscanner* package (Fig. S2A). Out of the total set of HIV reads obtained from all samples, 1.2% of reads were blacklisted (median 6 reads per sample, mean 16 reads). As expected, the majority of contaminant reads were found in samples that had very few total HIV reads (Fig. S2B). To validate the blacklisting procedure, we looked at reads within the *pol* gene, which contains the majority of drug resistance mutations. In “spike-in” experiments, where known fractions of contaminant reads were introduced and then recovered, “phyloscanner clean” correctly blacklisted 262 out of 274 contaminant reads, giving an overall sensitivity of 95.6%. The distribution of the spiked-in reads over the 50 samples is shown in Fig. S2C. Of the 291,815 noncontaminant reads, 291,742 were correctly identified, giving an overall specificity of over 99.9%.

The cost of implementing a high-throughput virus genomics system will vary by setting. In our laboratory in Oxford, the reagent, consumables, and labor costs of the entire assay, from frozen blood to final data, is approximately 30 GBP in 2020, 3 times lower than the WHO budget recommendations for HIV pol sequencing in low-income settings (24). Costs were reduced by concentrating total nucleic acid extractions to allow library preparations with one-quarter reagent volumes without losses in sequencing sensitivity (Fig. S1). With a throughput of 10,000 to 15,000 samples per year, 30 GBP per sample covers the salary of a UK technician processing 350 samples per week. Laboratory set-up costs (ground rental, equipment, and maintenance costs) are not included in this calculation.

### veSEQ-HIV yields quantitative viral loads.

Viral load is the concentration of virus in a sample and is usually measured with highly standardized and regulated clinical assays using quantitative PCR to amplify both the material to be tested and spiked internal standards of known viral load. Viral load tests are essential for rapid detection of resistance-associated treatment failure, but are expensive and not a part of routine care in many low-income countries.

**FIG 2:**
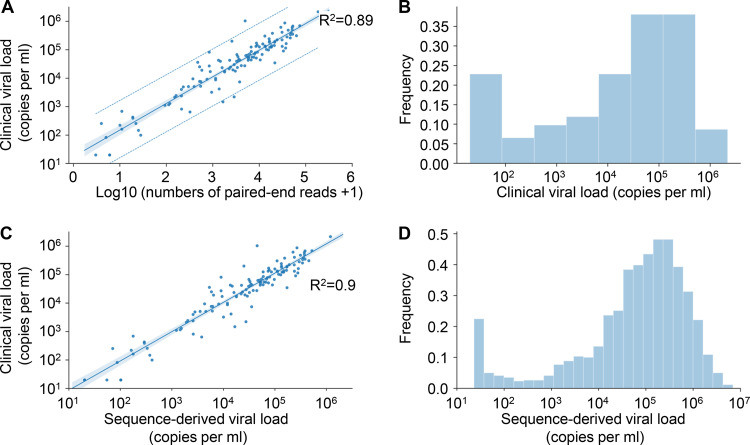
Viral load is calculated from the number of sequencing reads. (A) The data and linear regression model estimates for the viral load standards. The narrow shaded area is the 95% confidence interval for the regression curve, and the dashed lines are 95% prediction intervals for measurements. The mean squared error of prediction was 0.324 log_10_ copies/ml. (B) Distribution of independently measured clinical viral loads in a subset of 146 samples used to assess model performance. (C) Relationship between the clinical viral load and the sequence-derived viral load from the model shown in panel A for these 146 samples. (D) Frequency of sequence-derived viral load estimates for all 1,620 samples.

In a previous study of hepatitis C, we found that in contrast to amplicon-based sequencing, veSEQ was quantitative, in that total Illumina read-pairs correlated with clinical viral loads (25). To confirm that veSEQ was similarly quantitative for HIV, we performed both clinical viral load measurements and veSEQ-HIV sequencing on 146 specimens. Figure 2A shows the correlation between the routine clinically validated viral load and number of viral fragments recovered during sequencing, along with the *R*^2^ value (0.89). This correlation was robust over a wide range of viral loads (Fig. 2B) that includes the quantifiable limit of the clinical assay (<50 copies/ml).

The relationship between number of reads and viral load was linear on a log-log scale with a slope of 0.83. This corresponds to some nonlinearity on a linear scale, consistent with some loss of information at high viral loads, possibly due to saturation effects or erroneous bioinformatic-compression of distinct reads into single “deduplicated” reads (which is expected, by chance, at very high sequencing depth). This does not affect the use of the number of viral fragments to infer viral loads, since the relationship is well described mathematically. We therefore defined “sequence-derived log viral load” as the linear transform of the log number of deduplicated sequence fragments (Fig. 2C). The lower limit of detection was approximately 50 copies/ml. We calculated the sequence-derived viral load for all sequenced samples using this transformation and characterized the population distribution (Fig. 2D). This distribution was bimodal, with the minor peak at very low viral load, corresponding to individuals with HIV read counts below the quantifiable limit of conventional assays. In line with procedures used to calibrate clinical viral load assays, a serial dilution of inactivated cultured virus was included in each run to ensure the quality of the assay, guard against batch effects, and quantify rates of contamination between samples.

### veSEQ-HIV is unbiased with respect to viral genotype.

**FIG 3:**
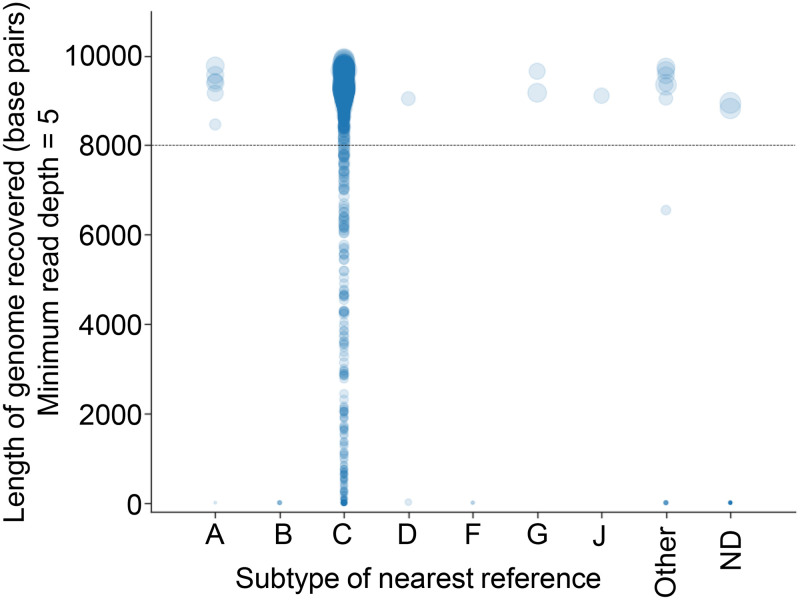
veSEQ-HIV is both sensitive and specific. Figure shows the length of recovered HIV genome for all sequenced samples. We consider a position in the genome to be accurately determined when the read depth is at least five. The category “Other” consists of potential intersubtype recombinants. Quantitative standards (HXB2, subtype B) are included in all sequencing runs, but are not displayed in this analysis.

Specificity for all known HIV subtypes circulating in Zambia was achieved using a probe-based, rather than a primer-based, amplification step (Fig. 3). HIV subtypes were inferred by sequence similarity to HIV reference genomes from the Los Alamos HIV database or by using the REGA HIV-1 subtyping tool (26). The predominant subtype was C, for which 86% (1,282/1,498) of samples yielded complete genomes. Eighteen nonsubtype C complete genomes included subtypes A (A1 and A2), D, G, and J, as well as the subtype B standards, demonstrating good probe affinity across HIV diversity. Given that partial genotypes are relatively harder to genotype correctly, it was unsurprising that 68% of ungenotyped sequences were incomplete (13/19), and those that were complete had features suggesting recombination.

### Assay sensitivity and associations between viral load, read depth, and genome coverage.

One of our aims was to ensure that veSEQ-HIV generated whole HIV genomes for the majority of samples within the range of viral loads observed in this population. The length of the recovered consensus sequence depends on the minimum read depth required to make a consensus call at each genomic position. We defined “read depth” as the number of mapped reads covering each position in the genome after removal of PCR duplicates. The point at which reads consistently matched the sample consensus saturated at a depth of five reads; we took this as our threshold for reliably inferring a consensus (Fig. S3). This may be a conservative estimate given that sequencing uninfected plasma (a negative control included in every run) resulted in no HIV reads after our multistage removal of contamination artifacts. However, we sought to produce not only accurate whole-genome consensus sequences, but also sufficient read depth for analyses of within-host diversity and characterization of low-frequency drug resistance mutations.

**FIG 4:**
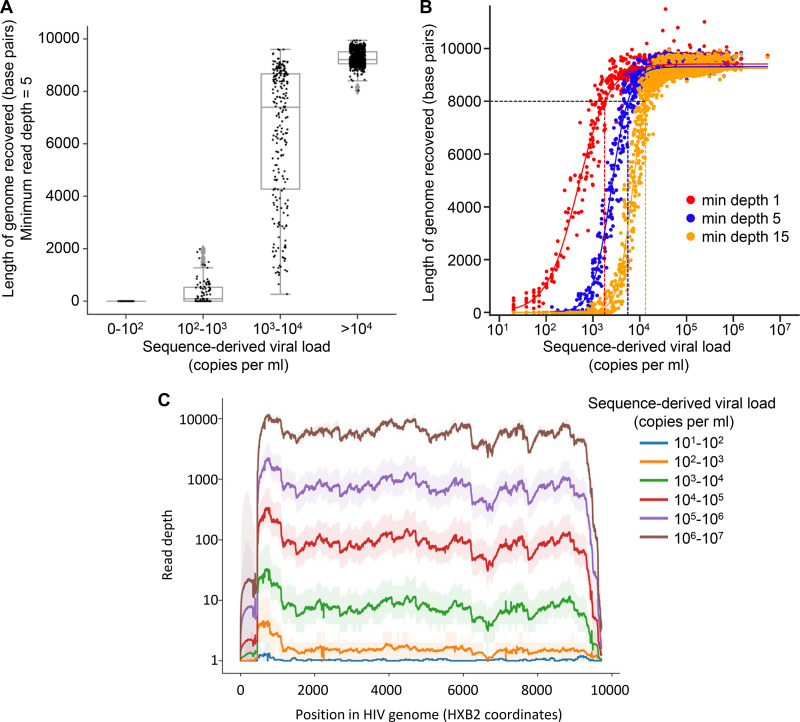
Sequencing success is influenced by viral load. (A) The length of the HIV genomes reconstructed by *shiver* software, from paired-end Illumina reads, stratified by log viral load, showed reproducible whole-genome coverage for samples with sequence inferred viral loads of >4 log_10_ copies/ml and near-complete coverage for the majority of samples with VL between 3 and 4 log_10_ copies/ml. (B) The viral loads at which genome coverage exceed 8 kb with minimum depth thresholds of 1 read, 5 reads, and 15 reads (after removal of PCR duplicates) are shown by the intercepts of curves fitted using a sigmoid function. (C) The median (thick lines) and 95^th^ percentile range (ribbons) of read-depth observed across the genome are shown for all samples, grouped by sequence-derived viral load.

Whole HIV genomes, defined as having a sequence length over 8,000 bp with a minimum read depth of five deduplicated reads, were obtained from all 1,204 samples with a viral load greater than 10,000 copies/ml and from 1,297/1,424 samples (91%) with a viral load greater than 1,000 (Table 1). The lowest viral load for which a whole genome was obtained was 4,300 copies/ml and 97% of samples above this threshold produced a whole genome (Fig. 4A). The majority of commercially available HIV-genotyping tests require a viral load of over 1,000 copies/ml. In this data set, 6% of samples had viral loads within the range of 1,000 to 4,300 copies/ml; at this range, the average length of genome covered was 4,172 bp (Fig. 4A).

**TABLE 1:** Numbers of samples processed using the sequencing pipeline and near-full genomes obtained (>8,000 bp), stratified by sequence-derived HIV-1 viral load (VL)

VL range (sequence derived)	Samples sequenced	Near-full-length genome
<10^2^	126	0
10^2^–10^3^	68	0
10^3^–10^4^	220	93
>10^4^	1,204	1,204

Higher viral loads in general resulted in higher read depth and therefore in greater coverage across the genome. Figure 4B shows the dependence of this success rate on sequence-derived viral load in more detail. Sigmoid functions (fit to the data with least-squares) indicate the viral load thresholds above which at least 8,000-bp genomes tend to be recovered: these are between 1,000 and 10,000 copies/ml, depending on the desired depth of reads supporting the consensus. Partial genomes were frequently obtained from samples with viral loads between 100 and 1,000 copies/ml (Fig. 4B).

The patterns of read depth were reproducible between individuals, with similar patterns of high and low coverage across the genome (Fig. 4C). Importantly, we did not observe a drop-off in coverage below five reads to be systematically associated with particular parts of the genome (Fig. S4).

### veSEQ-HIV provides drug resistance information on consensus and minority variant levels.

**FIG 5:**
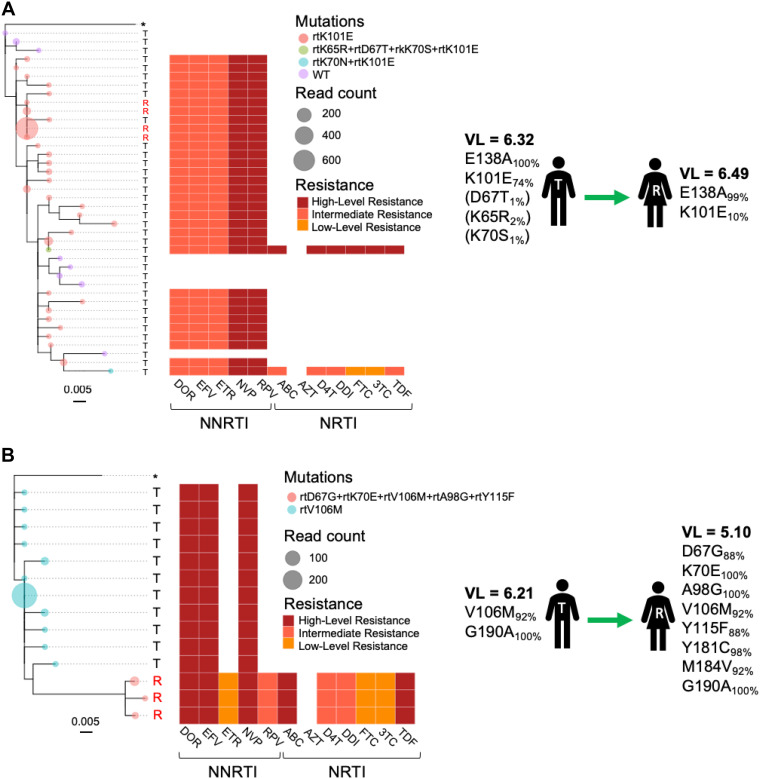
Within-host phylogenetic trees of Illumina reads spanning drug resistance sites in *pol. Phyloscanner* software performs ancestral state reconstructions of phylogenetic trees generated from Illumina reads in “windows” across the genome in order to identify pairs consisting of transmitters (T) and recipients (R). Phylogenetic trees of reads spanning drug resistance mutations sites in *pol* are shown for two inferred transmission pairs (A and B). Tree tips (circles) are colored by the combinations of drug resistance mutations observed for each unique taxon and scaled to total read counts within each taxa (after removal of PCR duplicates). Heatmaps report the predicted drug susceptibilities for each read using the Stanford HIVdb classification. Sequence-derived viral loads (log 10 RNA copies/ml) and the complete list of resistance mutations with associated frequencies, observed across entire genomes, are shown for each individual. Mutations observed at frequencies below 5% are shown in parentheses.

The quantitative nature of the veSEQ-HIV pipeline and its ability to identify and remove contamination artifacts are useful properties for characterizing drug resistance mutations at low frequency. In accordance with previously published guidance on generating drug resistance inferences from next-generation sequence data (27), we implemented a simple algorithm, based on the *HIVdb* classification system (Stanford, US), to predict overall susceptibilities to antiretroviral drugs from the resistance mutations identified on individuals reads, after cleaning with *phyloscanner*. A novel output of this approach was a detailed description of all mutations and combinations of mutations linked to within-host phylogenetic information that *phyloscanner* uses to infer transmission. Figure 5 provides representative examples of two transmission pairs, for which the direction of transmission had been systematically determined from ancestral-state reconstructions of multiple phylogenies of reads, performed in sliding windows across the genomes. Figure 5A depicts an example where a subclade of virus carrying the NNRTI resistance mutation K101E was transmitted to a female recipient. In the same transmission pair, subpopulations of wild-type susceptible virus and dual-class NRTI/NNRTI resistant virus (K65R/D67T/K70S/K101E and K70N/K101E) were not transmitted to the recipient, probably because they were detected in the transmitter at low frequency (<5%). In another transmission pair (Fig. 5B), V106M and G190A mutations were detected in the female recipient along with a number of additional mutations (D67G, K70E, A98G, Y115F, Y181C, and M184V) that were not found in the male transmitter, suggesting these additional mutations were acquired after the inferred transmission event. Consistent with this finding, the female recipient reported prior knowledge of her HIV-positive status and previous use of ART, although she was not on treatment at the time of sampling. Both individuals were sampled within 2 months of each other, and in the same health care facility.

## DISCUSSION

We have developed, optimized, and validated veSEQ-HIV, a fast, robust, cost-effective, and high-throughput laboratory and computational process for recovering complete HIV genomes, estimating viral load, detecting ART drug resistance mutations, and constructing transmission networks. The method has been shown to work with 1,620 genetically diverse samples collected from 10 Zambian clinics participating in HPTN 071-2 (PopART phylogenetics), producing whole genomes from >90% of samples with viral loads of >1,000 copies/ml. The assay works with residual plasma taken from routine CD4 cell count testing obtained in field laboratory conditions, without introducing undue contamination or degradation of the samples or the need for additional blood draws.

Our method has several advantages over previous high-throughput approaches (9). First, our probes were designed using an algorithm proven to tolerate levels of virus diversity even greater than that observed for HIV (e.g., HCV) (12), and are therefore expected to be unbiased with respect to the range of HIV variants commonly found in the region. Abbott Laboratories has recently reported on a similar method, developed in parallel to ours, which they show works across a wider panel of reference genomes (28, 29). Second, because our quantitative method minimizes the biases involved in PCR and computationally controls for contamination, our estimates of the frequency of minority genetic variants are likely to be more robust. Third, veSEQ is cost-effective. In our laboratory in Oxford, the reagent and consumables cost of the entire assay, from frozen blood to final data, is approximately 30 GBP in 2020, less than a fifth of the cost of the 2015 WHO budget for generating a full-genome sequence and viral load result (24). Our costing includes a technician salary, but not the initial costs of setting up a laboratory (equipment etc.)

The detection and quantification limits of veSEQ-HIV are comparable with those of clinical viral load assays (40 to 100 copies/ml), and inclusion of reference standards shows quantification is reproducible between runs. Sequencing from direct virus-PCR was previously shown to be less quantitative than veSEQ because of PCR saturation effects and because, unlike veSEQ, template resampling cannot be corrected with bioinformatic methods (for example, *PICARD MarkDuplicates*). Sequencing viral amplicons can be made quantitative with unique molecular identifiers (UMIs) that barcode single cDNA templates (30). In future work, we will evaluate whether addition of UMIs offers any additional benefit to the quantitativity and data quality of veSEQ-HIV. Here, we report an *R*^2^ value of 0.89 in a comparison with the Roche AmpliPrep TaqMan system, which is well within range of reported *R*^2^ values between commonly used clinical viral loads (0.80 to 0.94) (31). Additionally, *phyloscanner clean* provides a solution for “decontaminating” NGS data by removing low-frequency artifacts, such as index misassignments and PCR recombinants.

The throughput of veSEQ-HIV is suitable for large-scale public health applications. In our research setting, a single technician is able to process 360 samples per week. Routine combination testing to provide information on viral suppression, drug resistance, and transmission in near real time is feasible with veSEQ-HIV. This could prove useful as drug resistance surveillance is scaled up to guide and monitor new interventions, including preexposure prophylaxis, long-acting antiviral drugs, and alternative treatment regimens. High-resolution characterization of transmission events could augment precision public health programs and focused responses to local outbreaks. However, we caution that patient groups should be regularly consulted on the ethical use of this technology, to provide maximum benefit while minimizing risks to individuals (32).

There remain important limitations to our approach. While we have validated our methods to minimize contamination and provide quality control tools to detect mix-ups as quickly as possible, the risk of large-scale mix-ups increases with higher throughput. This should be mitigated with sample barcoding and sample tracking. Second, veSEQ-HIV is not licensed for clinical viral load, genotyping, or drug resistance testing. However, as part of the HPTN-078 study, drug resistance mutations detected by veSEQ-HIV were concordant with those detected by the FDA-accredited HIV genotyping test, ViroSeq. This study also validated viral load estimates against the Abbot RealTime Assay and found that veSEQ-HIV obtained complete drug resistance information 93.3% of the time in samples with viral loads of > 5000 RNA copies per ml (33).

The veSEQ-HIV protocol is tuned for high-throughput applications, and so is ideally suited for laboratories that process a large number of samples. Capital investments are modest, and the protocol is simple for technicians to adopt. However, maintenance and supply issues could be problematic in low- and middle-income countries, where the need is greatest. In such settings, centralized laboratory infrastructure could serve a number of districts. The computational component of the method is currently optimized for our local cluster infrastructure and will be streamlined and made platform-independent. Our current aim is for clinical accreditation of a complete laboratory and bioinformatics pipeline, operated by a single technician, data/lab manager, and clinical microbiologist, with remotely provided technical support, training, and quality assurance.

Future areas for improvement might include increasing automation, reducing initial capital expenditure costs, and reducing the reliance on regular supply chains of consumables. We did not explore the extent to which the bait capture step could be shortened or simplified; such improvements would further simplify the implementation of our method and would be needed to achieve a high-throughput protocol that could turn around sequence data in a single working day. Extending the length of individual sequences to capture whole viral haplotypes would improve applications in epidemiology and pathogenesis research.

The method can easily be adapted to study other RNA viruses, panels of RNA viruses, and even DNA and RNA viruses together, without loss of sensitivity (34) (preprint). Sequencing several pathogens simultaneously is achievable at minimal increased cost.

In summary, veSEQ-HIV is a cost-saving high-throughput protocol that, with current technologies, produces a sequence-derived viral load, a high-resolution drug resistance genotype, and data that can be used to provide highly granular insights into HIV epidemiology. The method has proven robust to field conditions in Zambia and carries no additional testing burden for patients. Sequencing will provide insights into the outcome of the HPTN 071 PopART trial (35) and, in our view, should be routinely performed in epidemiological and intervention studies of pathogenic viruses.

## Supplementary Material

Supplemental file 1
