# Supplementary material for: A Comprehensive Genomics Solution for HIV Surveillance and Clinical Monitoring in Low-Income Settings
Source: J Clin Microbiol. 2020 Sep 22;58(10):e00382-20. doi: 10.1128/JCM.00382-20 (PMC7512176; doi:10.1128/JCM.00382-20)
Supplement: Supplemental file 1 [file JCM.00382-20-s0001.pdf]

A comprehensive genomics solution for HIV surveillance and clinical monitoring in low income settings – Supplementary Material

Supplementary Table 1: Step-by-step protocol

**Laboratory**	
1	Viral particle lysis using chaotropic guanidine thiocyanate and total nucleic acid extraction using magnetised silica (easyMAG, bioMérieux).
2	RNA concentration and sample volume reduction using magnetised silica beads (RNAClean XP, Beckman Coulter).
3	Synthesis of libraries in low volume reactions, with low-temperature RNA denaturation and the Switching Mechanism At the 5' end of RNA Template (SMARTer) technology [1] to convert RNA to double stranded Illumina libraries within a single tube reaction (Clontech).
4	Double-indexing of sequencing libraries using indexed primers to reduce risks of index miss-assigned reads and false minority variants being generated by template switching. PCR cycles were minimized to reduce PCR duplicates, and to minimise short-read biasing.
5	Pooling libraries by equal volume, rather than by equal mass reduces hands-on time.
6	Size selection of pooled libraries using a stringent cleanup with magnetised silica beads
7	Bait capture of virus sequences using a panel of oligonucleotide probes designed to capture the expected diversity of HIV in sub-Saharan Africa.
8	Parallel library production and sequencing of 384 libraries on a HiSeq Rapid instrument set to produce 250 nt paired-end sequences within a single batch.
**Computational**	
1	Remove unwanted information and contaminants from raw sequencing output files. (Kraken [2])
2	Read trimming, quality control (minimum length 80bp; Trimmomatic [3])
3	Contig assembly (SPAdes [4], metaSPAdes [5]) and mapping (shiver [6], Kallisto [7])
3	Infer a sequence-derived viral load from numbers of Illumina read-pairs collected for each specimen (method described herein)
4	Infer consensus genotypes, minority variants and minority haplotypes.
5	Infer transmission chains, with quantified statistical support for links and direction of transmission. (phyloscanner [8])
6	Infer drug resistance, both at the consensus and minority haplotype level (HIVdb and drmSEQ ; Fogel et al. 2020. JAC in press).

Figure S1

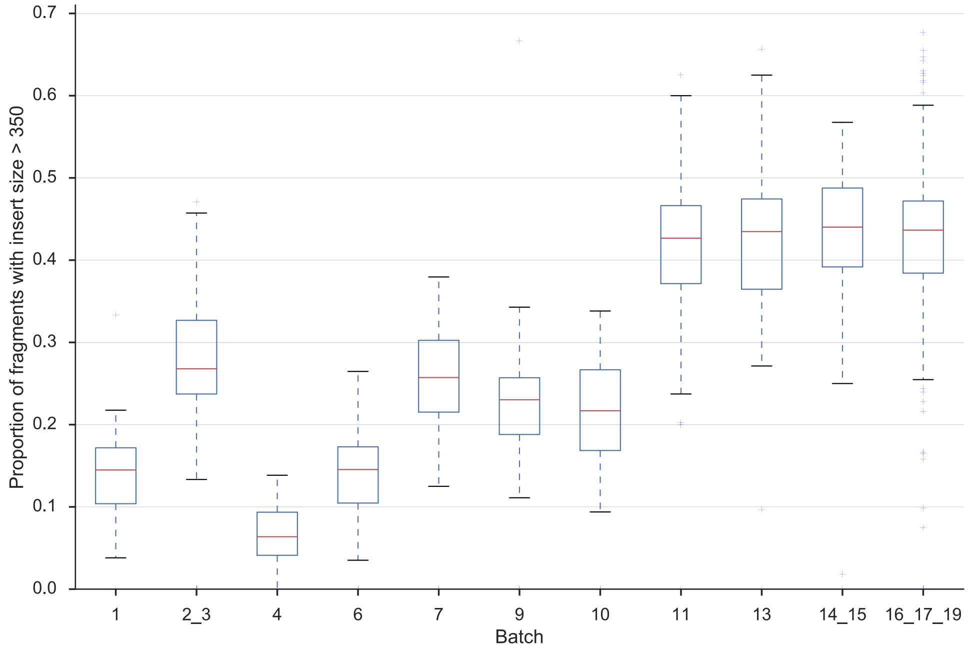

Figure S1: Optimisation of the proportion of sequenced fragments longer than 350 bp.

Batches are presented in chronological order, spanning a period of 18 months. The SMARTer protocol was introduced with batch 7; bead-based size selection was introduced at batch 11; reagent volumes were scaled down for batch 16_17_19 with no detrimental effect on the proportion of all de-duplicated fragments greater than 350 bp in length.

Figure S2

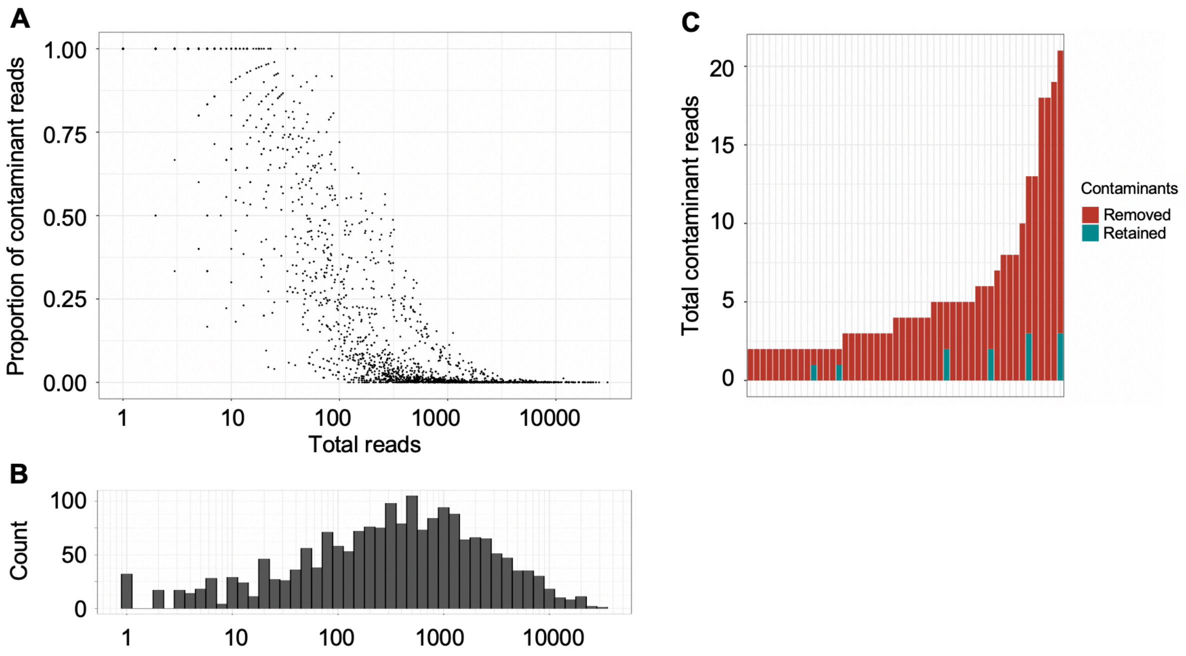

Figure S2: Contaminating reads are removed by blacklisting. **A.** Scatter plot of log10 of total number of reads assessed by *phyloscanner* in each sequenced sample, versus the proportion of reads identified as probable contaminants. Only read pairs that fully spanned the 250 bp windows were analysed. **B.** Corresponding density of *phyloscanner*-analysed reads in each sample, binned by log10 range. **C.** *Phyloscanner's* ability to accurately identify and remove contaminant reads was tested on a subset of 50 samples with at least 2,000 reads in pol that were deliberately contaminated by replacing 0.1% of reads from each dataset with the same amount from a separate dataset. Each column represents one of the 50 samples. sorted by number of artificially introduced contaminants. Red: reads correctly identified as contaminants. Turquoise: reads not identified as contaminants.

Figure S3

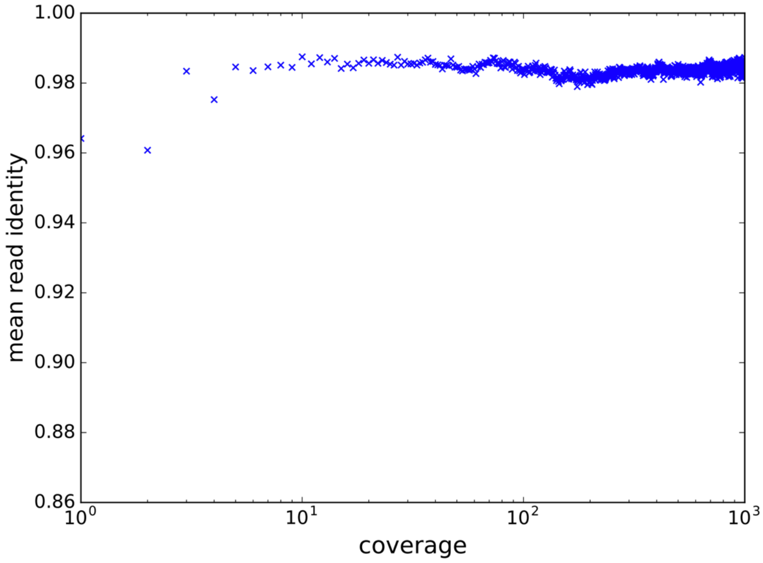

Figure S3: Sequence similarity between reads and consensus sequence by read depth.

Figure S4

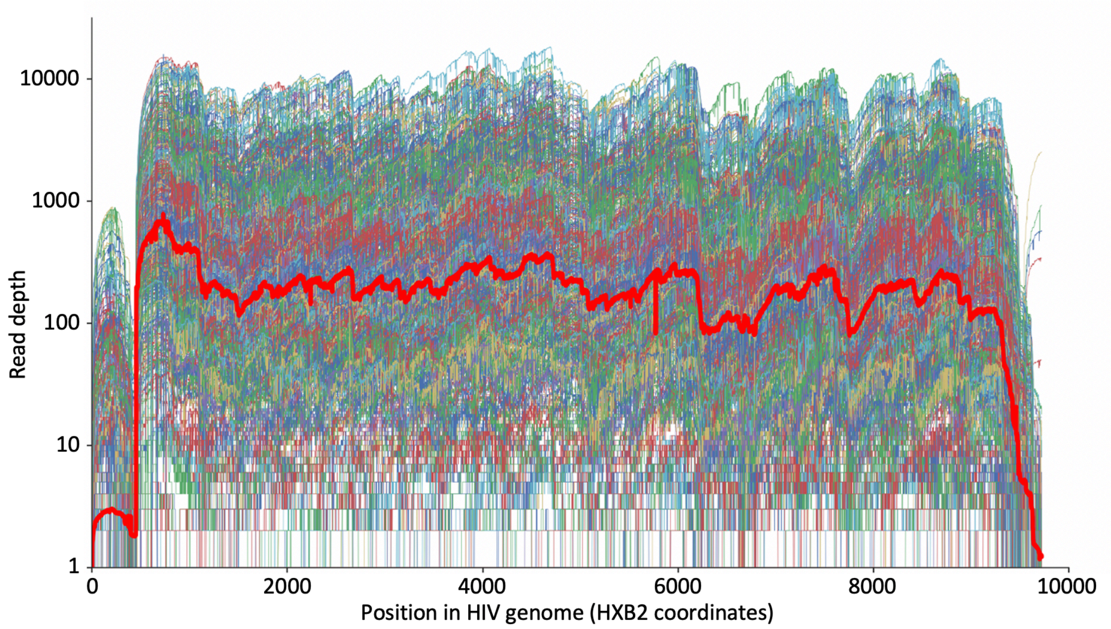

Figure S4: Read depth across the HIV genome for all samples in a single representative batch. Each coloured line corresponds to a single sample. The thick red line indicates the overall geometric mean for the batch.

Supplementary material references

[1] YY Zhu, EM Machleder, A Chenchik, R Li, PD Siebert. 2001. Reverse transcriptase template switching: a SMART approach for full-length cDNA library construction. Biotechniques 30:892–897.

[2] DE Wood, SL Salzberg. 2014. Kraken: ultrafast metagenomic sequence classification using exact alignments. Genome Biology 15:R46. URL 10.1186/gb-2014-15-3-r46

[3] AM Bolger, M Lohse, B Usadel. 2014. Trimmomatic: a flexible trimmer for Illumina sequence data. Bioinformatics 30:2114–2120.

[4] A Bankevich, S Nurk, D Antipov, AA Gurevich, M Dvorkin, AS Kulikov, VM Lesin, S I Nikolenko, S Pham, AD Prjibelski, AV Pyshkin, AV Sirotkin, N Vyahhi, G Tesler, M A Alekseyev, PA Pevzner. 2012. SPAdes: a new genome assembly algorithm and its applications to single-cell sequencing. J Comput Biol 19:455–477.

[5] S Nurk, D Meleshko, A Korobeynikov, PA Pevzner. 2017. metaSPAdes: a new versatile metagenomic assembler. Genome Res 27:824–834.

[6] C Wymant, F Blanquart, T Golubchik, A Gall, M Bakker, D Bezemer, NJ Croucher, M Hall, M Hillebregt, SH Ong, O Ratmann, J Albert, N Bannert, J Fellay, K Fransen, A Gourlay, MK Grabowski, B Gunsenheimer-Bartmeyer, HF Günthard, P Kivelä, R Kouyos, O Laeyendecker, K Liitsola, L Meyer, K Porter, M Ristola, A van Sighem, B Berkhout, M Cornelissen, P Kellam, P Reiss, C Fraser, BEEHIVE Consortium. 2018. Easy and accurate reconstruction of whole HIV genomes from shortread sequence data with shiver. Virus Evol 4:vey007.

[7] NL Bray, H Pimentel, P Melsted, L. Pachter. 2016. Near-optimal probabilistic RNA-seq quantification. Nat Biotechnol 34:525–527.

[8] C Wymant, M Hall, O Ratmann, D Bonsall, T Golubchik, M de Cesare, A Gall, M Cornelissen, C Fraser, STOP-HCV Cosortium, The Maela Pneumococcal Collaboration, The BEEHIVE collaboration. 2018. PHYLOSCANNER: Inferring Transmission from Within and Between-Host Pathogen Genetic Diversity. Mol Biol Evol 35:719-733
